# Comprehensive toxicity profile of the 4AC-4THP neoadjuvant regimen in HER2-positive breast cancer: a multicenter real-world study in Vietnam

**DOI:** 10.3389/fonc.2026.1678865

**Published:** 2026-03-09

**Authors:** Anh Dinh Tran, Le Huy Trinh, Tuan Anh Pham

**Affiliations:** 1 Department of Oncology, Hanoi Medical University, Hanoi, Vietnam; 2 Oncology Center, Hanoi Medical University Hospital, Hanoi Medical University, Hanoi, Vietnam; 3 Vietnam National Cancer Hospital, Hanoi, Vietnam

**Keywords:** 4AC-4THP, cardiotoxicity, HER2-positive breast cancer, neoadjuvant chemotherapy, real-world data, Vietnam

## Abstract

**Background:**

The 4AC-4THP regimen—comprising doxorubicin and cyclophosphamide followed by docetaxel, trastuzumab, and pertuzumab—has been widely adopted as neoadjuvant treatment for HER2-positive breast cancer. While efficacy data are well established, real-world safety data remain limited, particularly in Southeast Asian populations.

**Objective:**

To assess the comprehensive toxicity profile of the 4AC-4THP regimen, including both cardiac and non-cardiac adverse events, in Vietnamese patients with HER2-positive early-stage breast cancer.

**Methods:**

We conducted a retrospective multicenter study involving 52 women with stage II–III HER2-positive breast cancer treated with the 4AC-4THP neoadjuvant regimen at three oncology centers in Northern Vietnam between January 2020 and October 2024. Cardiotoxicity was assessed by serial echocardiography. Non-cardiac adverse events were graded according to CTCAE v5.0.

**Results:**

No patients developed symptomatic heart failure or experienced a decline in left ventricular ejection fraction (LVEF) below 50%. Subclinical LVEF reduction was observed in 78.8% of patients, with a mean decline of 8.05%, mostly during the anthracycline phase. Hematologic toxicities included neutropenia (42.3%, with 19.2% grade 3–4), anemia (46.2%), and thrombocytopenia (19.2%). Non-hematologic toxicities included fatigue/anorexia (76.9%), oral mucositis (67.3%), alopecia (100%), peripheral neuropathy (48.1%), and diarrhea (9.6%). All toxicities were manageable; no treatment-related deaths or discontinuations were reported.

**Conclusion:**

The 4AC-4THP neoadjuvant regimen in HER2-positive breast cancer showed acceptable tolerability in Vietnamese patients, especially among younger, low-comorbidity Asian populations. Subclinical cardiac dysfunction was common but mild and manageable. These findings provide further support for its use in neoadjuvant settings with appropriate monitoring strategies, especially in real-world clinical settings with variable access to monitoring resources.

## Introduction

Breast cancer remains the most prevalent malignancy among women worldwide. According to GLOBOCAN 2022 estimates, breast cancer is the most common malignancy among women in Vietnam, with approximately 24,562 new cases and 9,345 deaths reported annually, representing the highest cancer incidence and mortality burden among Vietnamese women (1). Approximately 15–20% of all breast cancers overexpress the human epidermal growth factor receptor 2 (HER2), which is associated with aggressive tumor behavior and poor prognosis if left untreated (2). However, the advent of HER2-targeted therapies such as trastuzumab and pertuzumab has significantly improved outcomes, especially in early-stage disease when used in neoadjuvant settings (3).

The neoadjuvant treatment approach enables tumor downstaging, increased breast conservation rates, and the potential for *in vivo* assessment of treatment response. Among the most commonly used regimens for HER2-positive breast cancer is a sequential protocol combining anthracyclines (doxorubicin and cyclophosphamide, AC) followed by taxane with dual anti-HER2 blockade (trastuzumab and pertuzumab), known as the 4AC-4THP regimen (4).

While this regimen is associated with high pathological complete response (pCR) rates, its safety profile warrants careful monitoring. Both anthracyclines and trastuzumab are independently associated with cardiotoxicity. In addition, hematologic and gastrointestinal adverse events are frequently reported. Although clinical trials have characterized the safety of this regimen, real-world data from low- and middle-income countries (LMICs) remain limited (5).

In low- and middle-income countries (LMICs) such as Vietnam, access to anti-HER2 therapies has improved over recent years through national insurance coverage and institutional support. However, resource constraints persist in terms of reimbursement policies, regional disparities in healthcare delivery, and variability in access to specialized monitoring and supportive care, rather than complete unavailability of targeted agents. These factors, along with demographic and potential genetic differences, may influence the real-world toxicity profile of intensive neoadjuvant regimens such as 4AC-4THP.

This study aimed to comprehensively evaluate the toxicity profile—especially cardiotoxicity—of the 4AC-4THP regimen in Vietnamese women with early-stage HER2-positive breast cancer treated in a real-world, multicenter setting.

## Materials and methods

### Study design

This retrospective observational study was conducted at three major oncology treatment centers in Northern Vietnam: Hanoi Medical University Hospital, Vietnam National Cancer Hospital (K Hospital), and Hanoi Oncology Hospital. Institutional ethics approval was obtained from all participating sites. Medical records from January 2020 to October 2024 were reviewed. The study complied with the 1964 Helsinki Declaration and was approved by the Ethics Committee of Hanoi Medical University (IRB-VN 01001), along with the ethical approval certificate number 934/GCN-HĐĐĐNCYSH-ĐHYHN.

### Eligible participants and materials

A total of 52 female patients with stage II or III HER2-positive breast cancer who received neoadjuvant chemotherapy with the sequential 4AC-4THP regimen were included in the analysis.

### Inclusion criteria

• Female patients aged ≥18 years
• Histologically confirmed HER2-positive invasive breast carcinoma (IHC 3+ or FISH-amplified).
• TNM staging (AJCC 8th edition) was determined using clinical examination and standard imaging modalities. No distant metastases at diagnosis (M0).
• Administration of the 4AC-4THP neoadjuvant treatment regimen.
• Available baseline and follow-up echocardiographic data

### Exclusion criteria

• Pre-existing symptomatic cardiovascular disease or LVEF <55% at baseline
• Incomplete treatment or missing toxicity data
• Discontinuation of therapy for non-toxicity reasons (Patients who discontinued neoadjuvant treatment for reasons unrelated to treatment-related toxicity (e.g., personal decision, financial constraints, loss to follow-up) were excluded from the final analysis).

Regarding the treatment regimen, patients received neoadjuvant therapy based on the 4AC-4THP regimen. The 4AC regimen consisted of Doxorubicin at a dose of 60 mg/m^2^ and Cyclophosphamide at 600 mg/m^2^, administered every 2 weeks (dose-dense) or every 3 weeks with granulocyte colony-stimulating factor support. The 4THP regimen included Trastuzumab at 8 mg/kg for the first cycle and 6 mg/kg from the second cycle, combined with Pertuzumab at 840 mg for the first cycle and 420 mg from the second cycle, along with a taxane (Docetaxel at 75–100 mg/m^2^ every 3 weeks or Paclitaxel at 80 mg/m^2^ on days 1, 8, and 15 of every 3-week cycle, or Paclitaxel at 175 mg/m^2^ every 2 weeks).

### Monitoring and assessments

Cardiac function was assessed using transthoracic echocardiography according to routine clinical practice at each participating center. Echocardiographic evaluations were performed at baseline prior to treatment initiation, after completion of the AC phase, and after completion of the THP phase, when available.

Left ventricular ejection fraction (LVEF) measurements were obtained as part of standard clinical care. Echocardiographic assessments were not centrally reviewed and were not performed in a blinded manner, reflecting the real-world design of the study.

Adverse events were recorded based on medical records and classified according to the Common Terminology Criteria for Adverse Events (CTCAE) v5.0, including:

• Cardiotoxicity
• Hematologic toxicity (anemia, neutropenia, thrombocytopenia)
• Gastrointestinal toxicity (nausea, vomiting, diarrhea)
• Dermatologic reactions
• Hepatic and renal function abnormalities (6)

### Definition of cardiotoxicity

Cardiac function was assessed using echocardiography at baseline, after completion of the anthracycline-containing regimen (AC), and after completion of the taxane–trastuzumab–pertuzumab (THP) regimen. Cardiotoxicity was defined based on criteria adapted from the CTCAE v5.0 and TRYPHAENA trial:

-Symptomatic cardiac dysfunction was defined as the presence of clinical signs and symptoms consistent with New York Heart Association (NYHA) class III or IV heart failure, associated with a decrease in left ventricular ejection fraction (LVEF) of ≥10 percentage points from baseline to a value below 50%.
-Asymptomatic cardiac dysfunction was defined as a reduction in LVEF of ≥10 percentage points from baseline to a value <50% in the absence of heart failure symptoms (7).
-Subclinical LVEF reduction was defined as an absolute decrease in LVEF of ≥10 percentage points from baseline with preserved LVEF ≥50%, without clinical symptoms of heart failure. This category was used to capture mild, asymptomatic changes in cardiac function that do not meet criteria for overt or CTCAE-defined cardiotoxicity but may have potential prognostic relevance.

Patients were closely monitored throughout neoadjuvant treatment. No prophylactic cardioprotective agents were administered.

### Statistical analysis

Descriptive statistics were used for baseline characteristics and adverse event rates.

• Continuous variables were expressed as mean ± standard deviation (SD) or median (interquartile range [IQR])
• Categorical variables were summarized as frequencies and percentages.

Given the relatively small sample size, 95% confidence intervals (CIs) for key proportions were calculated using the exact binomial (Clopper–Pearson) method.

Subgroup comparisons (e.g., by age group, comorbidities, baseline LVEF) were performed using Chi-square or Fisher’s exact tests for categorical variables, and t-tests or Mann-Whitney U tests for continuous variables as appropriate. A p-value <0.05 was considered statistically significant. Statistical analyses were performed using SPSS version 26.0. Missing data were handled using complete-case analysis, and no data imputation was performed due to the retrospective nature of the study. Given the descriptive objective of the analysis and the limited sample size, no adjustments for multiple comparisons were applied.

## Results

### Patients and treatment

There were a total of 52 patients diagnosed with HER2-positive breast cancer at stages IIA–IIIC who received treatment from January 2016 to October 2024 at Hanoi Medical University Hospital, Vietnam National Cancer Hospital, and Hanoi Oncology Hospital. These patients met the established inclusion criteria and were included in the analysis. The median age of the cohort was 41 years (range, 26–67 years), and the median tumor size was 37.5 mm. Of these 52 patients, 42 (80.8%) were premenopausal, while 10 (19.2%) were postmenopausal. Furthermore, 22 patients (42.3%) exhibited hormone receptor-positive status, and 1 patient’s HER2 status was confirmed via *in-situ* hybridization (ISH) testing. For age-based analyses, patients were categorized into younger (≤40 years) and older (>40 years) groups, consistent with commonly used definitions of young-onset breast cancer in Asian populations. The characteristics of the study population are summarized in **Table 1**.

**Table 1:** Baseline clinicopathological characteristics of patients treated with neoadjuvant 4AC-4THP (n = 52).

Characteristics		Number (n=52)	Percentage (%)
*Age*	<40	21	40.4
	≥40	31	59.6
*Menopausal status*	Menopausal	10	19.2
	Pre-Menopausal	42	80.8
*Cardiovascular or metabolic comorbidities*	Hypertension	3	5.8
	Diabetes	2	3.8
	Chronic kidney disease	1	1.9
	Current or past smoking history	1	1.9
*Primary stage*	IIA	17	32.7
	IIB	13	25.0
	IIIA	14	26.9
	IIIB	3	5.8
	IIIC	5	9.6
*Histopathology*	Invasive breast carcinoma – NST	40	76.9
	Other invasive breast carcinomas	12	23.1
*Tumor grade*	2	34	65.4
	3	18	34.6
*Hormon receptor status*	Positive (ER and/or PR positive)	22	42.3
	Negative	30	57.7
*HER2 status*	IHC (+++)	51	98.1
	IHC (++) – Dual-ISH (+)	1	1.9
*Ki-67*	< 20%	2	3.8
	≥ 20%	50	96.2
*AC regimen*	AC dose-dense (2-week)	48	92.3
	AC 3-week	4	7.7
*THP regimen*	Docetaxel – HP	26	50.0
	Paclitaxel weekly – HP	7	13.5
	Paclitaxel 2-week – HP	19	36.5
*Pathological response*	pCR	40	76.9
	No pCR	12	23.1

ER, estrogen receptor; PR, progesterol receptor; AC, anthracyclin, cyclophosphamide; THP, taxane, trastuzumab, pertuzumab; pCR, pathological complete response.

The pathological complete response (pCR) rate for both the primary tumor and axillary lymph nodes was 76.9% (40 out of 52).

### Cardiotoxicity

Among the 52 patients who completed the 4AC-4THP neoadjuvant regimen, no cases of symptomatic heart failure were observed during or after treatment. However, echocardiographic monitoring revealed subclinical declines in cardiac function in a significant proportion of patients. Overall, approximately 80% of patients experienced a decrease in left ventricular ejection fraction (LVEF) during treatment.

Importantly, none of the patients had a reduction in LVEF below 50%, and no patients required discontinuation of anti-HER2 therapy due to cardiotoxicity. The mean reduction in LVEF across the cohort was 8.05%, with a more pronounced decrease occurring after the anthracycline-based (AC) phase (approximately 6.1%) compared to the taxane-trastuzumab-pertuzumab (THP) phase (approximately 1.95%).

Echocardiography was performed at baseline, after completion of the 4AC phase, after completion of the THP phase, and prior to surgery. No clinical cardiac events were observed during the study period. All LVEF declines were asymptomatic, and recovery of LVEF was observed in most cases during follow-up.

There was no statistically significant difference in the incidence of subclinical LVEF decline between premenopausal and postmenopausal patients (p > 0.05). Consistently, age-based analysis showed that patients aged ≥40 years were not at increased risk of subclinical cardiac dysfunction compared with younger patients. Cardiotoxicity events observed in patients receiving the 4AC-4THP neoadjuvant regimen are summarized in **Table 2**.

**Table 2:** Cardiotoxicity in patients receiving the 4AC-4THP neoadjuvant regimen.

Cardiac parameters	Number of patients (n=52)	Percentage (%)	
Symptomatic heart failure	0	0.0%	
Any decrease in LVEF	41	78.8% (95% CI: 65.3–88.9%))	
LVEF < 50% or a decline of ≥10 percentage points	0	0.0%	
Mean reduction in LVEF (overall)	–	8.05% (mean)	
Mean reduction after AC phase	–	6.10% (mean)	
Mean reduction after THP phase	–	1.95% (mean)	
Discontinuation of anti-HER2 therapy due to cardiotoxicity	0	0.0%	
LVEF decline (any degree) – Postmenopausal (n = 10)	8	80.0%	p = 0.92
LVEF decline (any degree) – Premenopausal (n = 42)	33	78,6%	
LVEF decline (any degree) – Age <40 (n = 21)	16	76.2%	p = 0.75
LVEF decline (any degree) – Age ≥40(n = 31)	25	80.6%	

*LVEF*, Left Ventricular Ejection Fraction. Percentages are presented with exact binomial 95% confidence intervals due to the small sample size.

### Non-cardiotoxicities

## Discussion

This multicenter retrospective study provides a comprehensive safety profile of the 4AC-4THP neoadjuvant regimen in 52 Vietnamese patients with stage II–III HER2-positive breast cancer. The regimen was feasible in routine clinical practice, and no cases of symptomatic heart failure were reported. However, a high rate of subclinical cardiotoxicity and notable hematologic and non-hematologic adverse events were observed.

Cardiotoxicity remains a major concern in the treatment of HER2-positive breast cancer, particularly with the sequential use of anthracyclines and anti-HER2 therapy. In our study, although no patient developed symptomatic heart failure, a high proportion (78.8%) experienced subclinical LVEF reduction, defined as an asymptomatic ≥10% decline from baseline with preserved LVEF ≥50%. The mean absolute LVEF reduction was approximately 8%, and most of the decline occurred after the AC phase (6%) rather than during the THP phase (2%), suggesting a predominant role of anthracyclines in early subclinical cardiac changes.

Compared with the TRYPHAENA trial, which reported subclinical LVEF decline rates of 9.6%–11.4%, the incidence observed in our cohort was substantially higher (7). This difference should not be interpreted as inconsistency in treatment effect, but rather as a reflection of fundamental differences between randomized clinical trials and real-world practice. Patients enrolled in pivotal trials were highly selected and generally excluded those with baseline cardiovascular risk factors, whereas our real-world population likely included patients with a broader range of comorbidities and baseline cardiac vulnerability. In addition, routine clinical monitoring may detect mild, asymptomatic LVEF changes more frequently due to closer or more frequent assessments outside rigid trial protocols.

Furthermore, variations in anthracycline dose intensity, cumulative exposure, and the absence of standardized cardioprotective strategies and variability in access to cardio-oncology services in real-world settings may have contributed to the higher incidence of subclinical LVEF decline observed in our population. Importantly, while these LVEF reductions were largely asymptomatic and did not meet criteria for overt cardiotoxicity, accumulating evidence suggests that subclinical LVEF decline is not a benign finding, as it may be associated with incomplete recovery of cardiac function and an increased risk of subsequent symptomatic cardiotoxicity, particularly in patients receiving anthracycline-based and dual anti-HER2 therapy.

Nevertheless, the clinical and prognostic implications of these findings should be interpreted in light of the study’s limitations, including its retrospective design, small sample size, lack of a control group, and limited follow-up on LVEF recovery. Therefore, while our results underscore the importance of vigilant cardiac surveillance during anthracycline-containing neoadjuvant regimens in HER2-positive breast cancer, they should be generalized cautiously. Prospective studies with standardized cardiac monitoring protocols and longer follow-up are needed to better define the long-term significance of subclinical LVEF decline in real-world populations.

Other studies, including BCIRG-006 and NSABP B-31, have reported cardiotoxicity rates of 18–28% for LVEF drop and 2–4% for heart failure in anthracycline-trastuzumab arms (8, 9). Compared to these, our data suggest a low incidence of clinically overt cardiotoxicity despite a high rate of subclinical LVEF decline.

Traditional cardiovascular (CV) risk factors—including advanced age (generally ≥65 years, with particularly high risk at ≥75–80 years), hypertension, diabetes mellitus, chronic kidney disease, current or past smoking history, and obesity (BMI >30 kg/m^2^)—are well-recognized contributors to anthracycline- and trastuzumab-related cardiotoxicity (10–12). Additionally, pre-existing CV comorbidities such as heart failure, baseline left ventricular ejection fraction (LVEF) <50%, significant valvular heart disease, coronary artery disease or angina, and arrhythmias like atrial fibrillation further increase cardiotoxic risk (10–12). Prior exposure to other cardiotoxic cancer therapies, including anthracyclines, trastuzumab, and mediastinal or left chest radiotherapy, also compounds the risk. A previous randomized controlled trial reported cancer therapy–related cardiac dysfunction (CTRCD) in 8% of HER2-positive breast cancer patients treated with anthracyclines alone and in up to 27% of those treated with the combination of anthracyclines and trastuzumab (13).

In our study, however, the cumulative dose of doxorubicin administered was 240 mg/m^2^, which does not exceed the threshold dose (≥250 mg/m^2^) known to significantly increase cardiotoxicity risk (10). Moreover, the study population predominantly consisted of younger patients, with 80.8% (42/52) being premenopausal, no cases of obesity, and a very low prevalence of underlying cardiovascular or metabolic comorbidities. These factors may help explain why, despite a relatively high incidence of LVEF decline (approximately 80%**),** most decreases were around 10%, and no patient experienced an absolute LVEF drop below 50%, suggesting that cardiotoxicity in this cohort was largely mild and potentially reversible and may partly explain the absence of clinically overt cardiotoxicity.

A recent long-term follow-up study by Jin U (2025) and colleagues reported reassuring cardiac safety outcomes with dose-dense anthracycline regimens in patients with non-metastatic breast cancer. In their cohort, comprehensive cardiac assessments—including echocardiography, electrocardiography, and biomarker analysis conducted 7–10 years after treatment completion—showed preserved left ventricular systolic function and no significant differences in LVEF compared with conventional regimens, with only one case of heart failure observed in the conventional therapy group (14). These findings highlight that, with careful patient selection and structured cardiac monitoring, dose-dense anthracycline therapy can maintain long-term cardiac function in selected patients. This evidence supports the feasibility of dose-dense anthracycline-based neoadjuvant regimens such as AC in our study, particularly in younger patients with low baseline cardiovascular risk and systematic echocardiographic surveillance.

Beyond clinical predictors, emerging evidence suggests that genetic predisposition plays a role in anthracycline-related cardiotoxicity. Recent genome-wide studies in both pediatric and adult cancer survivors have identified multiple variants associated with susceptibility to CTRCD. These include alterations in genes involved in topoisomerase IIβ-mediated DNA damage, reactive oxygen species generation, iron metabolism, drug transport, and sarcomere integrity (15). Although genetic testing for cardiotoxicity risk stratification remains investigational, it may offer future opportunities for personalized risk assessment, especially in populations like ours where traditional CV risk factors are not prominent yet subclinical cardiotoxicity is still observable.

Collectively, these findings highlight the complex interplay between clinical, pharmacologic, and genetic factors in determining the risk of cardiotoxicity. In real-world clinical settings, regular echocardiographic surveillance and early identification of functional decline remain essential components of safe administration of anthracycline and HER2-targeted therapies. Although longitudinal data on LVEF recovery were not systematically available in this retrospective cohort, previous studies have shown that anthracycline- and trastuzumab-related subclinical LVEF decline may be partially or fully reversible in a substantial proportion of patients with early detection and appropriate management. However, persistent subclinical dysfunction has also been associated with increased long-term cardiovascular risk, underscoring the importance of continued surveillance.

Hematologic toxicity was common, with grade 3–4 neutropenia in 19.2% of patients, mainly during the AC dose-dense phase, anemia (grades 1–2) occurred in 46%, while thrombocytopenia was mild and observed in 20%. No treatment-related deaths due to hematologic toxicity were observed (**Table 3**). This aligns with previous data showing that dose-dense anthracycline regimens are associated with high neutropenia rates (up to 50–60%) without prophylactic G-CSF. However, a recent study in Korean patients with early-stage breast cancer demonstrated that dose-dense AC chemotherapy with pegteograstim support is both tolerable and safe in the Asian population, suggesting that with appropriate supportive care, hematologic toxicities can be effectively managed (16). These results are comparable to our findings in Vietnamese patients, further supporting the feasibility of this regimen in Asian cohorts.

**Table 3:** Non-Cardiotoxicities in patients receiving the 4AC-4THP neoadjuvant regimen (n=52).

Adverse event	Grade 1-2 (n,%)	Grade 3-4 (n(%,95%CI)	Total (n,%)	Notable characteristics
Neutropenia	12 (23.1%)	10 (19.2%; 95% CI: 9.6–32.5)	22 (42.3%; 95% CI: 28.8–56.8)	Mostly occurred during AC dose-dense (9/10 cases of G3–4)
Anemia	24 (46.2%)	0 (0%; 95% CI: 0.0–6.8)	24 (46.2%; 95% CI: 32.2–60.7)	
Thrombocytopenia	10 (19.2%)	0 (0%; 95% CI: 0.0–6.8)	10 (19.2%; 95% CI: 9.6–32.5)	
Fatigue, anorexia	40 (76.9%)	0 (0%; 95% CI: 0.0–6.8)	40 (76.9%; 95% CI: 63.2–87.0)	
Oral Mucositis	33 (63.5%)	2 (2.8%) (3.8%; 95% CI: 0.5–13.0)	35 (63.5%; 95% CI: 49.0–76.4)	Primarily during AC phase
Diarrhea	5 (9.6%)	0 (0%; 95% CI: 0.0–6.8)	5 (9.6%; 95% CI: 3.2–21.0)	
Peripheral neuropathy	25 (48.1%)	0 (0%; 95% CI: 0.0–6.8)	25 (48.1%; 95% CI: 34.0–62.4)	Almost related to paclitaxel use in THP regimen; all Grade 1–2
Infusion-related reaction (taxane)	2 (3.8%)	0 (0%; 95% CI: 0.0–6.8)	2 (3.8%; 95% CI: 0.5–13.0)	
Alopecia	52 (100%)	0 (0%; 95% CI: 0.0–6.8)	52 (100%)	Total alopecia in all patients

Adverse events were graded according to the Common Terminology Criteria for Adverse Events (CTCAE) version 5.0. Most non-cardiotoxicities were mild to moderate and manageable with supportive care. No treatment-related deaths or permanent discontinuation of therapy due to non-cardiotoxicity were reported. Percentages are presented with exact binomial 95% confidence intervals (Clopper–Pearson method) due to the small sample size.

Non-hematologic adverse events such as fatigue, alopecia, mucositis, and peripheral neuropathy were frequent but mostly grade 1–2. Neuropathy was notably more common in patients receiving paclitaxel-based THP, consistent with prior studies like CLEOPATRA and TRAIN-2 (17, 18).

The overall toxicity pattern in our study reflects that of previous trials but emphasizes that real-world patients may experience slightly higher adverse event rates due to broader inclusion criteria and comorbidities.

Our findings suggest that the 4AC-4THP regimen is effective and manageable in the Vietnamese setting, with appropriate monitoring for cardiotoxicity. Given the high rate of subclinical cardiac dysfunction, routine echocardiographic follow-up and early cardiology consultation are advisable.

The data reinforce the importance of tailoring neoadjuvant therapy based on individual patient characteristics, particularly in Asian populations. Future research should consider prospective designs and longer follow-up to assess long-term cardiac outcomes and recurrence-free survival.

This study is limited by its retrospective nature, moderate sample size, and lack of a control group. Cardiac biomarkers (e.g., troponin, NT-proBNP) were not routinely collected, limiting mechanistic insight into cardiotoxicity. Moreover, pCR and survival data were not the focus of this manuscript and warrant future analysis.

## Conclusion

In this real-world cohort, the 4AC-4THP regimen was associated with a high incidence of subclinical cardiac function decline and clinically relevant hematologic toxicities. While most adverse events were clinically manageable with appropriate monitoring and supportive care, these findings underscore the necessity of intensive cardiac monitoring and proactive toxicity management when this regimen is applied in routine clinical practice.

## Limitations

This study has several important limitations. First, its retrospective real-world design limits causal inference and is subject to selection and information bias. Second, the small sample size (N = 52) and prolonged accrual period restricted statistical power, precluding meaningful subgroup analyses or adjustment for treatment heterogeneity; therefore, exact binomial 95% confidence intervals were reported to reflect the uncertainty of toxicity estimates.

Third, cardiac monitoring was performed according to routine clinical practice rather than a standardized protocol, and echocardiographic assessments were not blinded and were conducted at predefined clinical milestones rather than uniform study time points. In addition, data on long-term recovery of left ventricular ejection fraction were limited, which may partly explain the high rate of subclinical LVEF decline observed compared with clinical trials.

Fourth, patients who discontinued treatment for non-toxicity-related reasons were excluded, which may have introduced selection bias and led to an underestimation of real-world intolerance. Finally, the absence of a concurrent anthracycline-free control group limits direct comparison with non-anthracycline regimens and warrants cautious interpretation of the findings.

## Data Availability

The raw data supporting the conclusions of this article will be made available by the authors, without undue reservation.

## References

[1] Cancer today. Available online at: https://gco.iarc.who.int/today/ (Accessed August 3, 2025).

[2] Breast cancer HER2 status | What is HER2 status? Available online at: https://www.cancer.org/cancer/types/breast-cancer/understanding-a-breast-cancer-diagnosis/breast-cancer-her2-status.html (Accessed August 3, 2025).

[3] Xu L Xie Y Gou Q Cai R Bao R Huang Y . HER2-targeted therapies for HER2-positive early-stage breast cancer: present and future. Front Pharmacol. (2024) 15:1446414. doi: 10.3389/fphar.2024.1446414, PMID: 39351085 PMC11439691

[4] Dowling GP Keelan S Toomey S Daly GR Hennessy BT Hill ADK . Review of the status of neoadjuvant therapy in HER2-positive breast cancer. Front Oncol. (2023) 13:1066007. doi: 10.3389/fonc.2023.1066007, PMID: 36793602 PMC9923093

[5] Wu YT Xu Z Zhang K Wu JS Li X Arshad B . Efficacy and cardiac safety of the concurrent use of trastuzumab and anthracycline-based neoadjuvant chemotherapy for HER2-positive breast cancer: a systematic review and meta-analysis. Ther Clin Risk Manage. (2018) 14:1789–97. doi: 10.2147/TCRM.S176214, PMID: 30310287 PMC6165855

[6] National Cancer Institute . Common Terminology Criteria for Adverse Events (CTCAE). (2018) Published October 15, 2018. Available online at: https://www.cancer.gov/research/resources (Accessed August 3, 2025).

[7] Schneeweiss A Chia S Hickish T Harvey V Eniu A Waldron-Lynch M . Long-term efficacy analysis of the randomised, phase II TRYPHAENA cardiac safety study: Evaluating pertuzumab and trastuzumab plus standard neoadjuvant anthracycline-containing and anthracycline-free chemotherapy regimens in patients with HER2-positive early breast cancer. Eur J Cancer Oxf Engl 1990. (2018) 89:27–35. doi: 10.1016/j.ejca.2017.10.021, PMID: 29223479

[8] Slamon D Eiermann W Robert N Pienkowski T Martin M Press M . Adjuvant trastuzumab in HER2-positive breast cancer. N Engl J Med. (2011) 365:1273–83. doi: 10.1056/NEJMoa0910383, PMID: 21991949 PMC3268553

[9] Perez EA Romond EH Suman VJ Jeong JH Davidson NE Geyer CE . Four-year follow-up of trastuzumab plus adjuvant chemotherapy for operable human epidermal growth factor receptor 2-positive breast cancer: joint analysis of data from NCCTG N9831 and NSABP B-31. J Clin Oncol Off J Am Soc Clin Oncol. (2011) 29:3366–73. doi: 10.1200/JCO.2011.35.0868, PMID: 21768458 PMC3164242

[10] Lyon AR López-Fernández T Couch LS Asteggiano R Aznar MC Bergler-Klein J . 2022 ESC Guidelines on cardio-oncology developed in collaboration with the European Hematology Association (EHA), the European Society for Therapeutic Radiology and Oncology (ESTRO) and the International Cardio-Oncology Society (IC-OS). Eur Heart J. (2022) 43:4229–361. doi: 10.1093/eurheartj/ehac244, PMID: 36017568

[11] Armenian SH Lacchetti C Barac A Carver J Constine LS Denduluri N . Prevention and monitoring of cardiac dysfunction in survivors of adult cancers: american society of clinical oncology clinical practice guideline. J Clin Oncol Off J Am Soc Clin Oncol. (2017) 35:893–911. doi: 10.1200/JCO.2016.70.5400, PMID: 27918725

[12] Barrett-Lee PJ Dixon JM Farrell C Jones A Leonard R Murray N . Expert opinion on the use of anthracyclines in patients with advanced breast cancer at cardiac risk. Ann Oncol Off J Eur Soc Med Oncol. (2009) 20:816–27. doi: 10.1093/annonc/mdn728, PMID: 19153118

[13] Slamon DJ Leyland-Jones B Shak S Fuchs H Paton V Bajamonde A . Use of chemotherapy plus a monoclonal antibody against HER2 for metastatic breast cancer that overexpresses HER2. N Engl J Med. (2001) 344:783–92. doi: 10.1056/NEJM200103153441101, PMID: 11248153

[14] Jin U . Revisiting the cardiac safety of dose-dense chemotherapy: encouraging outcomes from long-term follow-up. Int J Heart Fail. (2025) 7:107–9. doi: 10.36628/ijhf.2025.0028, PMID: 40519708 PMC12160050

[15] Al-Otaibi TK Weitzman B Tahir UA Asnani A . Genetics of anthracycline-associated cardiotoxicity. Front Cardiovasc Med. (2022) 9:867873. doi: 10.3389/fcvm.2022.867873, PMID: 35528837 PMC9068960

[16] Kim GM Kim JH Kim JH Cho YU Kim SI Park S . A phase II study to evaluate the safety and efficacy of pegteograstim in korean breast cancer patients receiving dose-dense doxorubicin/cyclophosphamide. Cancer Res Treat. (2018) 51:812–8. doi: 10.4143/crt.2018.383, PMID: 30235921 PMC6473288

[17] Swain SM Baselga J Kim SB Ro J Semiglazov V Campone M . Pertuzumab, trastuzumab, and docetaxel in HER2-positive metastatic breast cancer. N Engl J Med. (2015) 372:724–34. doi: 10.1056/NEJMoa1413513, PMID: 25693012 PMC5584549

[18] van Ramshorst MS van der Voort A van Werkhoven ED Mandjes IA Kemper I Dezentjé VO . Neoadjuvant chemotherapy with or without anthracyclines in the presence of dual HER2 blockade for HER2-positive breast cancer (TRAIN-2): a multicentre, open-label, randomised, phase 3 trial. Lancet Oncol. (2018) 19:1630–40. doi: 10.1016/S1470-2045(18)30570-9, PMID: 30413379

